# A Genetically Encoded Tag for Correlated Light and Electron Microscopy of Intact Cells, Tissues, and Organisms

**DOI:** 10.1371/journal.pbio.1001041

**Published:** 2011-04-05

**Authors:** Xiaokun Shu, Varda Lev-Ram, Thomas J. Deerinck, Yingchuan Qi, Ericka B. Ramko, Michael W. Davidson, Yishi Jin, Mark H. Ellisman, Roger Y. Tsien

**Affiliations:** 1 Howard Hughes Medical Institute, University of California at San Diego, La Jolla, California, United States of America; 2 Department of Pharmacology, University of California at San Diego, La Jolla, California, United States of America; 3 National Center for Microscopy and Imaging Research, Center for Research on Biological Systems, University of California at San Diego, La Jolla, California, United States of America; 4 Division of Biological Science, Section of Neurobiology, University of California at San Diego, La Jolla, California, United States of America; 5 National High Magnetic Field Laboratory and Department of Biological Science, The Florida State University, Tallahassee, Florida, United States of America; 6 Department of Neurosciences, University of California at San Diego, La Jolla, California, United States of America,; 7 Department of Chemistry and Biochemistry, University of California at San Diego, La Jolla, California, United States of America; University of Colorado, United States of America

## Abstract

Electron microscopy (EM) achieves the highest spatial resolution in protein localization, but specific protein EM labeling has lacked generally applicable genetically encoded tags for in situ visualization in cells and tissues. Here we introduce “miniSOG” (for mini Singlet Oxygen Generator), a fluorescent flavoprotein engineered from *Arabidopsis* phototropin 2. MiniSOG contains 106 amino acids, less than half the size of Green Fluorescent Protein. Illumination of miniSOG generates sufficient singlet oxygen to locally catalyze the polymerization of diaminobenzidine into an osmiophilic reaction product resolvable by EM. MiniSOG fusions to many well-characterized proteins localize correctly in mammalian cells, intact nematodes, and rodents, enabling correlated fluorescence and EM from large volumes of tissue after strong aldehyde fixation, without the need for exogenous ligands, probes, or destructive permeabilizing detergents. MiniSOG permits high quality ultrastructural preservation and 3-dimensional protein localization via electron tomography or serial section block face scanning electron microscopy. EM shows that miniSOG-tagged SynCAM1 is presynaptic in cultured cortical neurons, whereas miniSOG-tagged SynCAM2 is postsynaptic in culture and in intact mice. Thus SynCAM1 and SynCAM2 could be heterophilic partners. MiniSOG may do for EM what Green Fluorescent Protein did for fluorescence microscopy.

## Author Summary

Electron microscopy (EM) once revolutionized cell biology by revealing subcellular anatomy at resolutions of tens of nanometers, well below the diffraction limit of light microscopy. Over the past two decades, light microscopy has been revitalized by the development of spontaneously fluorescent proteins, which allow nearly any protein of interest to be specifically tagged by genetic fusion. EM has lacked comparable genetic tags that are generally applicable. Here, we introduce “miniSOG”, a small (106-residue) fluorescent flavoprotein that efficiently generates singlet oxygen when illuminated by blue light. In fixed tissue, photogenerated singlet oxygen locally polymerizes diaminobenzidine into a precipitate that is stainable with osmium and therefore can be readily imaged at high resolution by EM. Thus miniSOG is a versatile label for correlated light and electron microscopy of genetically tagged proteins in cells, tissues, and organisms including intact nematodes and mice. As a demonstration of miniSOG's capabilities, controversies about the localization of synaptic cell adhesion molecules are resolved by EM of miniSOG fusions in neuronal culture and intact mouse brain.

## Introduction

The most general techniques for imaging specific proteins within cells and organisms rely either on antibodies or genetic tags. EM is the standard technique for ultrastructural localization, but conventional EM immunolabeling remains challenging because of the need to develop high-affinity, high-selectivity antibodies that recognize cross-linked antigens, and because optimal preservation of ultrastructure and visibility of cellular landmarks requires strong fixation that hinders diffusibility of antibodies and gold particles. Thus the target proteins most easily labeled are those exposed at cut tissue surfaces. Replacement of bulky gold particles by eosin enables catalytic amplification via photooxidation of diaminobenzidine (DAB), but eosin-conjugated macromolecules still have limited diffusibility and need detergent permeabilization to enter cells [1]. Genetic labeling methods should overcome many of these shortcomings, just as fluorescent proteins have revolutionized light microscopic imaging in molecular and cell biology [2]. However, no analogous genetically encoded tag for EM contrast has yet proven widely applicable. Metallothionein has been proposed as a genetic tag that can noncatalytically incorporate cadmium or gold [3], but its main applications to intact cells have been to *Escherichia coli* conditioned to tolerate 0.2 mM CdCl_2_ for 18 h [4] or 10 mM AuCl for 3 h [4],[5]. Such high concentrations of heavy metal salts would not seem readily transferable to most multicellular organisms or their cells. Also many higher organisms express endogenous metallothionein, which would contribute background signals unless genetically deleted or knocked down [5]. Horseradish peroxidase can be a genetic label in the secretory pathway but is greatly limited by its requirements for tetramerization, glycosylation, and high Ca^2+^, so that it is not functional when expressed in the cytosol [6]. Furthermore, its DAB reaction product tends to diffuse from sites of enzymatic generation, resulting in poorer resolution than immunogold or the reaction product of photogenerated singlet oxygen (^1^O_2_, the metastable excited state of O_2_) with DAB [1],[7],[8]. The best previous genetically targetable generator of ^1^O_2_ was the biarsenical dye ReAsH, which binds to genetically appended or inserted tetracysteine motifs [9]. However, ReAsH has modest ^1^O_2_ quantum yield (0.024) (Figure S1), requires antidotes to prevent cell toxicity, needs careful precautions to reduce nonspecific background signal, and has been difficult to apply to multicellular tissues and organisms [10]. Although fluorescence photooxidation using GFP has been reported [11],[12], the ^1^O_2_ quantum yield of the naked GFP chromophore is extremely low (0.004), and the ^1^O_2_ quantum yield of the intact protein was yet lower and unquantifiable [13], presumably because the beta-barrel of the protein shields the chromophore from oxygen. The phototoxic fluorescent protein “Killer Red” [14] is now acknowledged not to work through ^1^O_2_ [15], and we have confirmed that its ^1^O_2_ quantum yield is negligible (Figure S1).

Here, we introduce miniSOG, a small, genetically encodable protein module that needs no exogenous cofactors to fluoresce and photogenerate ^1^O_2_ with a substantial quantum yield. MiniSOG provides major improvement in correlated light and electron microscopy in cells and multicellular organisms via photooxidation techniques.

## Results

### Structure-Based Design of MiniSOG

**Figure 1:**
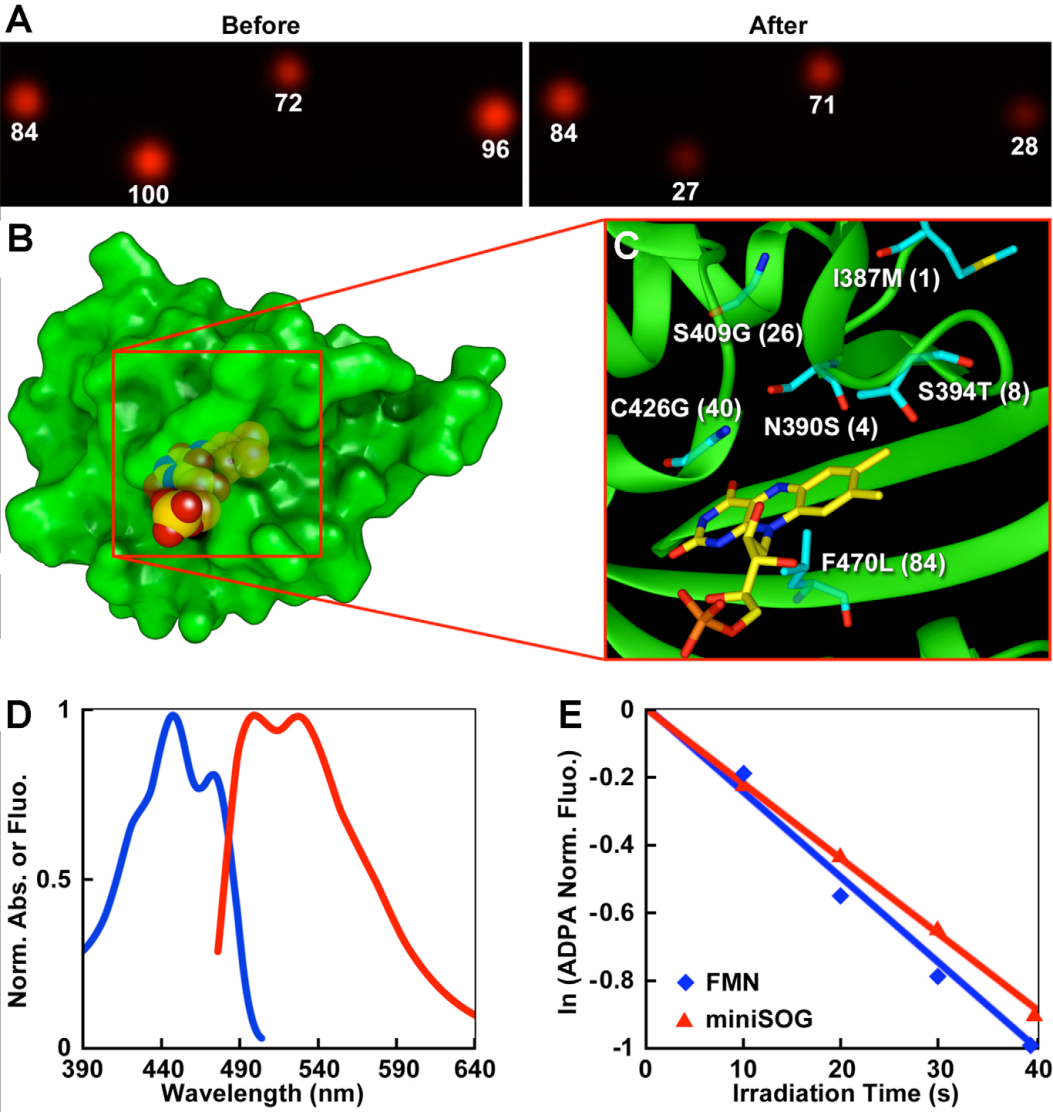
MiniSOG, a small and efficient singlet oxygen generator, is engineered from a blue light photoreceptor based on protein crystal structure. (A) Infrared fluorescence of *E. coli* colonies expressing the fusion proteins before and after irradiation (480±15 nm excitation). (B) Predicted structure of miniSOG by the Swiss-Model structure homology-modeling server [52]. (C) Mutations introduced into miniSOG compared to its parent. Numbers in bracket are based on miniSOG protein sequence. (D) Normalized absorbance (blue) and emission (red) spectra. (E) Degradation of ADPA by illumination of miniSOG (red) or free FMN (blue).

The LOV (light, oxygen, and voltage) domain of phototropin (a blue light photoreceptor) binds flavin mononucleotide (FMN) [16],[17], which by itself is an efficient singlet oxygen photosensitizer [18]. FMN is ubiquitous in cells and performs indispensable biological functions such as mitochondrial electron transport, fatty acid oxidation, and vitamin metabolism [19]. In phototropin, the excited state energy of FMN is consumed to form a covalent bond with a cysteine [20]. To divert this energy into ^1^O_2_ generation, we carried out saturation mutagenesis of the relevant cysteine (Cys426) of the LOV2 domain of *Arabidopsis thaliana* phototropin 2 (*AtPhot2*). To screen for optimal ^1^O_2_ production, these site-specific mutants were fused to an infrared fluorescent protein, IFP1.4, which is readily bleached by ^1^O_2_ (Figure S2) [21]. Colonies of *E. coli* expressing the fusion proteins were imaged in the IFP channel (ex 684/em708 nm) before and after blue light (488 nm) illumination (Figure 1A). Several colonies showed a decrease of IFP fluorescence from wild-type colonies and two with the largest decrease (∼70%) had the single site substitution of Cys426 to Gly. The small side chain of the glycine residue may provide space around the cofactor that would allow O_2_ close apposition to FMN for efficient energy transfer. To increase the brightness of the C426G mutant, we also performed saturation mutagenesis of other residues surrounding the chromophore binding site. DNA shuffling of the improved mutants plus random mutagenesis led to a new protein, miniSOG (106-residue) (Figure 1B and C, Figure S3), which absorbs maximally at 448 nm with a shoulder at 473 nm with extinction coefficients (16.7±0.7)×10^3^ and (13.6±0.5)×10^3^ M^−1^cm^−1^, respectively (Figure 1D). Excitation of miniSOG leads to green emission with two peaks at 500 and 528 nm (Figure 1D). The ^1^O_2_ quantum yield of miniSOG (0.47±0.05) was measured using anthracene-9,10-dipropionic acid (ADPA) as ^1^O_2_ sensor (Figure 1E) [22]. Free FMN was used as the standard for the measurement of ^1^O_2_ generation (quantum yield 0.51) [10].

MiniSOG was determined by light scattering to be monomeric in solution, with a molecular weight of 13.9±0.4 kDa, close to the theoretical value of 15.3 kDa. Absence of oligomerization was further supported by the good separation by gel filtration of miniSOG from its tandem dimer (td-miniSOG) (Figure S4). Mass spectrometry confirmed that the flavin cofactor is FMN (Figure S5). Equilibrium dialysis reported a dissociation constant of 170±8 pM (Table S2), similar to values for some flavoproteins (e.g. 260±60 pM for a flavodoxin [23]) and consistent with the crystal structures of LOV domains, which show FMN deeply buried inside the protein core [24]. Furthermore, overexpression of miniSOG in HEK293 cells caused the FMN content to increase ∼3-fold, presumably to keep miniSOG nearly saturated with FMN (Figures S6–S8), but caused no obvious toxicity in the absence of light (Table S1). Feedback pathways involving enzymes such as riboflavin kinase (EC 2.7.1.26) and FAD (flavin adenine dinucleotide) diphosphatase (EC 3.6.1.18) probably regulate intracellular FMN to titrate endogenous flavoproteins and miniSOG [25]. Riboflavin kinase phosphorylates riboflavin into FMN, while FAD diphosphatase catalyzes the production of FMN from FAD.

### Correct Localization of Well-Understood Proteins Tagged with MiniSOG in Tissue Culture Cells

**Figure 2:**
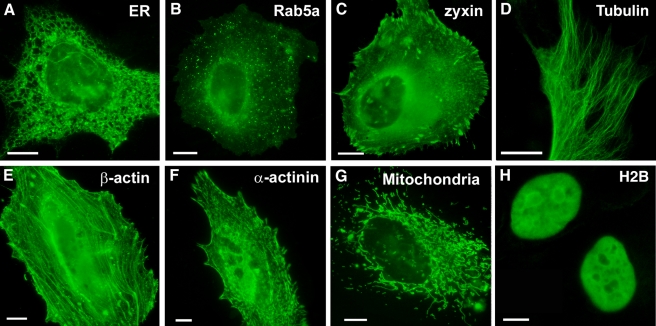
MiniSOG-labeled proteins and organelles exhibit correct localization at the light microscopic level. Confocal fluorescence images of miniSOG-targeted endoplasmic reticulum (A), Rab5a (B), zyxin (C), tubulin (D), β-actin (E), α-actinin (F), mitochondria (G), and histone 2B (H) in HeLa cells; scale bars, 10 µm.

**Figure 3:**
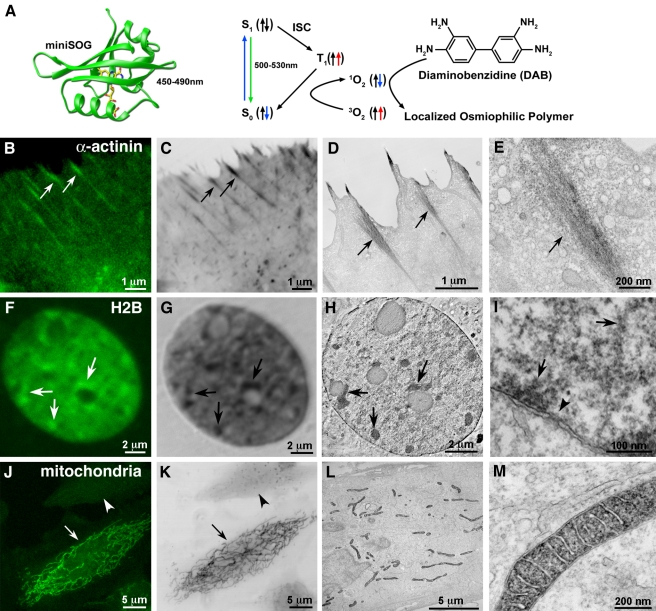
MiniSOG produces correlated fluorescence and EM contrast with correct localization of labeled proteins and organelles. (A) Schematic diagram of how miniSOG produces EM contrast upon blue-light illumination. Spin states are depicted by the arrows. ISC, intersystem crossing. Correlated confocal fluorescence (B,F,J), transmitted light (C,G,K), and electron microscopic (D,E,H,I,L,M) imaging of a variety of proteins. (B–E) HeLa cells expressing miniSOG labeled α-actinin. Arrows denote correlated structures. (F–I) Histone 2B. Panel H is a 3 nm thick computed slice from an electron tomogram. Panel I is a high magnification thin section electron micrograph showing labeled chromatin fibers near the nuclear envelope (arrows) and a nuclear pore (arrowhead). (J–M) Mitochondrial targeted miniSOG. Panels J and K show a confocal image prior to photooxidation and a transmitted light image following photooxidation, respectively. The differential contrast generated between a transfected (arrows) and non-transfected cell (arrowheads) is evident. Bars B–D, 1 micron; E, 200 nm; F–H, 2 microns; I, 100 nm; J–L, 5 microns; M, 200 nm.

We used the fluorescence from miniSOG fusion proteins to successfully localize a wide variety of proteins and organelles in cultured mammalian cells (Figure 2). Its green fluorescence, while modest compared to GFP (quantum yield of 0.37 versus 0.6), revealed that labeled components appeared to have correct localizations (Figure 2A–H). Figure 2A shows ER-targeted miniSOG, indicating that miniSOG can work within the secretory pathway. Figure 2B–F show Rab5a, zyxin, tubulin, β-actin, and α-actinin as examples of proteins tagged in cytosolic compartments. Mitochondrial targeting and nuclear histone 2B-fusions (Figure 2G,H) show that miniSOG expresses within those organelles. Using the fluorescence and photo-generated ^1^O_2_ from miniSOG for fluorescence photooxidation of DAB (Figure 3A), correlated confocal and EM imaging could be performed with several miniSOG fusion proteins (Figure 3B–E), producing excellent EM contrast, efficient labeling, and good preservation of ultrastructure.

#### α-Actinin

α-Actinin cross-links actin bundles and attaches actin filaments to focal adhesions (FA) [26]. EM images of stained miniSOG fusion proteins expressed in HeLa cells contained fibrous densities consistent with published observations associating α-actinin with actin bundles in the cell cortex adjacent to the plasma membrane FA-like structures (Figures 3B–E, S9C–D). The higher contrast between cells expressing miniSOG tagged α-actinin versus non-expressing cells is clearly evident in the cytosol in these electron micrographs (Figure S9A).

#### Histone 2B (H2B)

MiniSOG-tagged H2B revealed large-scale organizations of chromatin fibers in the perinucleolar and intranuclear regions [27] as imaged by confocal fluorescence, transmitted light after photooxidation, and correlated thin section and electron tomography (arrows, Figure 3F–H). The tomographic slice demonstrates the utility of miniSOG labeling for 3-dimensional EM analysis. Fibrillar chromatin structures near the nuclear envelope and nuclear pores were also observable at high resolution (arrows and arrowhead, respectively; Figure 3I). The H2B fusion seemed to have no deleterious effects when incorporated into chromosomes since H2B-miniSOG expressing cells can be found in several stages of mitosis (Figure S10).

#### Mitochondrial matrix

Mitochondria containing cytochrome C–targeted miniSOG fusions had well-preserved morphology of outer and inner membranes and cristae with a strong EM signal present in the mitochondrial matrix consistent with the targeting (Figure 3L and M). The contrast differential between mitochondria in cells expressing targeted miniSOG and photooxidized compared to adjacent cells not expressing miniSOG is apparent by both LM (Figure 2J and K) and EM (Figure S9B).

#### Connexin 43 (Cx43)

**Figure 4:**
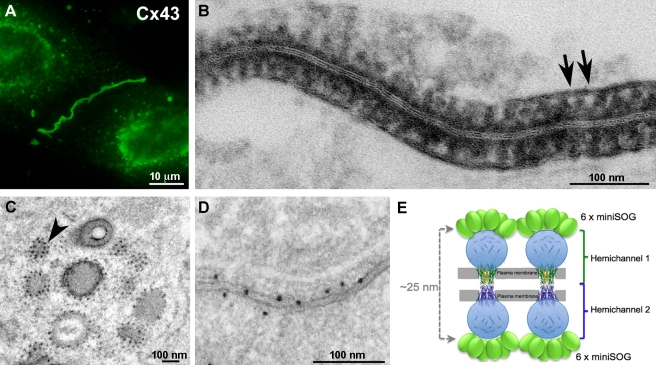
MiniSOG-tagged Cx43 forms gap junctions. (A) The green fluorescence of miniSOG reveals gap junctions and transporting vesicles. (B) Electron microscopy indicates negatively stained structures of appropriate size and spacing to be gap junction channels (arrows). (C) Studs on the membranes of trafficking vesicles suggest single connexons. The arrowhead points to two dots with a center-to-center distance ∼14 nm. (D) A high-quality immunogold image showing a randomly labeled fraction of densely packed Cx43 gap junctions. This figure is reproduced from Figure 4D of Gaietta et al. [9]. (E) A cartoon showing miniSOG-labeled Cx43 gap junctions. Bar A, 10 microns; B–D, 100 nm.

Cx43 forms gap junction channels. EM of the Cx43-miniSOG fusion showed densely stained DAB photooxidation reaction product outlining structures (Figure 4B) roughly corresponding in size to gap junction channels each composed of 12 connexins (six in each hemichannel). A cartoon (Figure 4E) based on the x-ray crystal structure of the transmembrane and extracellular domains of Cx26, which shares 46% sequence identity with that of Cx43 [28], and the NMR structure of the carboxy-terminal domain of Cx43 [29] is shown for interpretation of the EM. Furthermore, we speculate that the black dots studded on the outside of trafficking vesicles (black dots, Figure 4C) may represent single connexons [30]–[32]. As a comparison, EM of densely packed Cx43 gap junctions using immunogold showed much sparser, more random labeling (Figure 4D).

### Localization of MiniSOG in Tissues of Multicellular Organisms

#### C. elegans mitochondrial labeling

**Figure 5:**
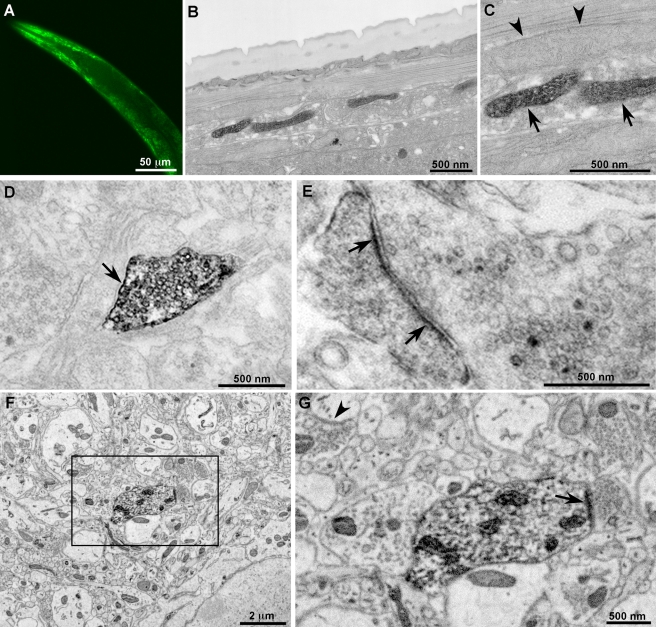
MiniSOG produces fluorescence and EM contrast in *C. elegans* and reveals previously unknown localization of synaptic cell adhesion molecules in mice. (A) Confocal fluorescence image of miniSOG targeted to the mitochondria in body wall muscles of *C. elegans*. (B–C) Thin section EM images of a portion of *C. elegans* showing a subset of labeled mitochondria in the body wall muscle (arrow) and adjacent unlabeled mitochondria in a different cell type (arrowheads). (D–E) Ultrastructural localization of miniSOG-labeled synaptic cell-adhesion molecules (SynCAMs) in cultured cortical neurons. (D) SynCAM1 fusion reveals uniform membrane labeling at the presynaptic apposition (arrow). (E) SynCAM2 fusion shows postsynaptic membrane labeling (pointed by arrow). Ultrastructural details including synaptic vesicles and nerve terminal substructure were well preserved in both (D) and (E). (F–G) Ultrastructural localization of miniSOG-labeled synaptic cell-adhesion molecule 2 (SynCAM2) in intact mouse brain. (A) A large area (∼14 µm × 14 µm) of one of the tissue sections imaged by serial block-face scanning electron microscopy. (B) Enlargement of the region boxed in (A) reveals postsynaptic membrane labeling (pointed by arrow) apposing a presynaptic bouton containing vesicles. Ultrastructural details including synaptic vesicles and membrane-bound structures of synapses were well preserved and easily recognizable (e.g. arrowhead in the upper left). Bar A, 50 microns; B–C, 500 nm; D–E, 500 nm; F, 2 microns; G, 500 nm.

We expressed miniSOG in the matrix of body wall muscle mitochondria using a cytochrome c targeting sequence in *C. elegans* to explore the usefulness of miniSOG for correlated fluorescence and EM in multicellular organisms. In transgenic worms the green fluorescence of miniSOG showed labeled mitochondria in body wall muscle cells (Figure 5A) while EM revealed a subset of stained mitochondria with well-preserved morphology (Figure 5B,C).

#### Pre- and post-synaptic localization of SynCAM1 and 2, respectively

To ascertain if miniSOG could reveal new molecular details of the organization of neuronal synapses, we expressed miniSOG attached to two isoforms of SynCAM to determine their locations in synapses of mouse neurons. SynCAMs are cell-adhesion molecules involved in synapse formation, maturation, and plasticity whose extensive expression throughout the brain suggests important functions [33]. SynCAMs play an important role in establishing and stabilizing synapses through Ca^2+^-independent interactions, in contrast to Ca^2+^-dependent neurexin-neuroligin interactions [34]. In spite of their recognized role in synapse assembly, the specific localization of SynCAMs had not been accomplished previously. A prior EM study suggested both pre- and post-synaptic membrane localization of SynCAM1 using antibodies raised against its C-terminus, but ambiguity remained because these antibodies cross-react with SynCAM2 and SynCAM3 [33],[34]. To overcome this limitation, we separately examined the synaptic distribution of SynCAM1 and SynCAM2 fusions to miniSOG, initially in cultured cortical neurons. SynCAM1-miniSOG was found only at presynaptic terminals, identified by the presence of synaptic vesicles, confirming a presynaptic localization (Figure 5A, Figure S10). This presynaptic targeting of SynCAM1-miniSOG was also observed in transfected single neurons forming synapses onto themselves in a micro-island culture system (Figure S11) [35], ruling out the possibility that postsynaptic neurons are more difficult to identify or transfect. In contrast, SynCAM2 localized to postsynaptic sites in cultured cortical neurons, identified by postsynaptic densities and by the opposition of these terminals to presynaptic boutons bearing synaptic vesicles (Figure 5B, Figure S12).

Next, we introduced these fusion proteins into prenatal mouse brains by *in utero* electroporation in order to study their localizations. Because neurons expressing miniSOG fusion proteins may be sparse, we turned to serial block-face scanning electron microscopy (SBFSEM), a relatively new method that facilitates large-scale 3–D reconstruction of tissue to help systematically find synapses from the few transfected neurons within the brains of young adults. The instrument consists of an ultramicrotome fitted within a backscatter-detector equipped scanning electron microscope. In an automated process, the ultramicrotome removes an ultra-thin section of tissue with an oscillating diamond knife and the region of interest is imaged. This sequence is repeated hundreds or thousands of times until the desired volume of tissue is traversed. This method potentially enables the reconstruction of microns to tenths of millimeters of volumes of tissue at a level of resolution better than that obtainable by light microscopy [36],[37]. However, optimal backscatter signal is dependent on very strong scattering from heavy metal stains. The photooxidation of MiniSOG generated a strongly osmiophilic reaction product that in combination with en bloc uranyl acetate staining provided a specific and strong backscatter electron signal, which confirmed that the fusion to SynCAM2 was postsynaptic in intact mouse brain (Figure 5). Thus, the combination of miniSOG fusion proteins and SBFSEM provides a method to correlate the location of specific molecules throughout large 3–D volumes and with good preservation of ultrastructure (Figure S13).

## Discussion

The successful localization of a variety of proteins by light and EM in cultured cells as well as mitochondria in *C. elegans* and SynCAM2 in intact mouse brain demonstrates the value of miniSOG for correlated light and EM localization of specific proteins in cells and multicellular organisms. MiniSOG is advantageous over conventional immuno-gold staining because the protein of interest is genetically tagged before fixation and all subsequent components (O_2_, DAB, and OsO_4_) are small molecules that easily permeate tissues. Tissues or cells can be fixed using established methods for good preservation of ultrastructure without concern for retention of antigenicity. Thus, permeabilizing detergents such as Triton X-100 that degrade membranes to facilitate the diffusion of bulky antibodies and secondary labels are unnecessary. This is demonstrated by the well-preserved ultrastructure in SynCAM-miniSOG labeled mice where unlabeled synapses (arrowhead), nonsynaptic plasma membrane, and synaptic vesicles are clearly observed (Figure 5). Such landmarks were essential to assign the precise location of the SynCAMs. While super-resolution fluorescence techniques [38]–[40] could provide improved localizations, each landmark of interest would need to be labeled with fluorophores emitting at different color.

MiniSOG probes have several advantages over other correlated LM/EM probes. MiniSOG needs no exogenous cofactors and produces ^1^O_2_ with about 20 times higher quantum efficiency than ReAsH on a tetracysteine motif. Therefore, miniSOG photooxidation has considerably better sensitivity and lower background than ReAsH labeling. MiniSOG is much smaller than GFP, and unlike GFP can mature and become fluorescent in the absence of O_2_. GFP-based photooxidation is very difficult due to its extremely low ^1^O_2_ quantum yield [13]. Genetically encoded horseradish peroxidase is tetrameric and far larger than GFP, only becomes functional inside the secretory pathway [6], and produces relatively diffuse precipitates [1],[7],[8]. Metallothionein fusions would seem most appropriate for purified macromolecules [3], because imaging of intact cells requires them to survive prolonged incubation in high concentrations of Cd^2+^ or Au^+^ [4],[5] and not to express endogenous metallothionein.

Our results with miniSOG fusions demonstrate that SynCAM1 and SynCAM2 are localized to pre- and post-synaptic membranes, respectively, and these observations are consistent with the reported strong heterophilic interaction between SynCAM1 and SynCAM2 in the formation of trans-synaptic structures [41]. The presynaptic membrane localization of SynCAM1 is also consistent with the recent report that SynCAM1 is expressed in growth cones in the early developmental stages of mouse brain and is involved in shaping the growth cones and the assembly of axo-dendritic contact [41]. Analogous trans-synaptic pairs include neurexin/neuroligin [42], EphrinB/EphB, and netrinG/netrin-G ligand (NGL). New synaptic proteins continue to be reported, such as leucine rich repeat transmembrane proteins (LRRTMs), NGL-3, and leukocyte common antigen-related (LAR) [43],[44]. The large variety of these molecules may be necessary to establish and support the great diversity of neuronal synapses; dissecting their locations within synapses will be a complex task.

As demonstrated here, our miniSOG-based photooxidation technique provides a method to determine the detailed distribution of these and other important macromolecules. In combination with SBFSEM, miniSOG fusion proteins should find wide applications in the ultrastructural localization of proteins, including 3-d reconstruction of neuronal circuits by large scale automated SBFSEM to mark cells of interest and trace them across large numbers of sections (Figure S13) [37]. Additionally, a logical next step will be to further enhance the preservation of cellular ultrastructure in these types of specimens by combining chemical fixation and high pressure freezing [45] with photooxidation using miniSOG.

Spatiotemporally controlled local photogeneration of ^1^O_2_ should also be useful for rapidly inactivating proteins of interest [46], reporting protein proximities over tens of nanometers [47] by ^1^O_2_ transfer from a SOG to a ^1^O_2_ sensitive fluorescent protein (e.g. IFP1.4) and ablating cells by photodynamic damage. Thus, further development and application of miniSOG using ^1^O_2_ generation should greatly expand its utility in imaging and functional studies.

## Materials and Methods

### Gene Synthesis, Mutagenesis, and Screening

A gene encoding LOV2 domain of *Phototropin 2* with codons optimized for *E. coli* was synthesized by overlap extension PCR [48]. Genetic libraries were constructed by saturation and random mutagenesis and DNA shuffling [21]. Mutants were fused to IFP1.4 by overlap extension PCR and cloned into a modified pBAD vector containing the heme oxygenase-1 gene from cyanobacteria [21]. Libraries were expressed in *E. coli* strain TOP10 and screened by imaging the agar plates with colonies in the IFP channel before and after blue light illumination [21]. Protein purification and spectroscopic characterization experiments were done as described [49].

### Chimera Construction

DNA encoding miniSOG with codons optimized for mammals was synthesized by overlap extension PCR [48]. MiniSOG fusions were cloned into pcDNA3.1 vector. HEK293 and HeLa cells were transfected with miniSOG or chimera cDNAs using Fugene, then imaged 24–48 h later. Cultured cortical neurons were transfected by Amaxa electroporation (Lonza AG, Germany) and imaged 1–2 wk later.

### Fluorescence Imaging, Photooxidation, and EM Preparation of Transfected Cultured Cells

Transfected cells cultured on glass bottom culture dishes (P35G-0-14-C, MatTek Corp., Ashland, MA) were fixed with 2% glutaraldehyde (Electron Microscopy Sciences, Hatfield, PA) in pH 7.4 0.1 M sodium cacodylate buffer (Ted Pella Inc., Redding, CA) for 30–60 min, rinsed several times in chilled buffer, and treated for 30 min in blocking buffer (50 mM glycine, 10 mM KCN, and 5 mM aminotriazole) to reduce nonspecific background reaction of diaminobenzidine (DAB). Confocal images were taken with minimum exposure using a BioRad MRC-1024 inverted confocal microscope or similar inverted fluorescence microscope to identify transfected cells and for correlative light microscopic imaging. Detailed protocols for performing fluorescence photooxidation of DAB have been published [2],[6]. It is important to use an inverted microscope to ensure direct open access to the DAB solution. An objective of numerical aperture ≥0.7 is desirable to maximize illumination intensity. For photooxidation, diaminobenzidine tetrahydrochloride (Sigma-Aldrich, St. Louis, MO) was freshly diluted to 1 mg/ml in 0.1 M sodium cacodylate buffer, pH 7.4, filtered through a 0.22 micron syringe filter (Millipore), and placed on ice and added to the cells. The region of interest was identified by the fluorescence and an image recorded with care not to bleach the area. A small tube attached to an oxygen tank was placed near the top of the dish and a stream of pure oxygen was gently blown continuously over the top of the solution. Alternately, the DAB solution on ice was bubbled with oxygen and the solution in the dish refreshed every few minutes. The samples were then illuminated using a standard FITC filter set (EX470/40, DM510, BA520) with intense light from a 150W xenon lamp. Illumination was stopped as soon as a very light brown reaction product began to appear in place of the green fluorescence as monitored by transmitted light (typically 2–10 min, depending on the initial fluorescence intensity, the brightness of the illumination, and the optics used). Care was taken to avoid overreacting the samples, as this can lead to overstaining and the degradation of ultrastructure in the region of photooxidation. Multiple areas on a single dish could be reacted if the solution was refreshed every few minutes. The cells were then removed from the microscope and washed in chilled buffer (5×2 min) and post-fixed in 1% osmium tetroxide (Electron Microscopy Sciences) in 0.1 M sodium cacodylate buffer for 30 min on ice. Cells were washed in chilled buffer twice and rinsed in distilled water, then *en bloc* stained with 2% aqueous uranyl acetate (Ted Pella Inc.) for 1 h to overnight at 4°C. The samples were then dehydrated in a cold graded ethanol series (20%, 50%, 70%, 90%, 100%, 100%) 2 min each, rinsed once in room temperature anhydrous ethanol, and infiltrated in Durcupan ACM resin (Electron Microscopy Sciences) using 1∶1 anhydrous ethanol and resin for 30 min, then 100% resin 2×1 h, then into fresh resin and polymerized in a vacuum oven at 60°C for 48 h.

### Preparation of *C. elegans*

Transgenic worms were made by injection of cDNAs of mitochondrially targeted miniSOG driven by *myo-3* promoter at 50 ng/µl. The worms were chemically fixed with 2% glutaraldehyde, washed, and blocked as described above. The cuticle was sharply cut to allow diffusion of DAB into the inner body for photooxidation. After confocal imaging and fluorescence photooxidation, the worms were processed for EM imaging as described above.

### Preparation, Fluorescence Imaging, and Photooxidation of Mouse Brain

Endotoxin-free DNA (∼3 µg) of the SynCAM2-miniSOG fusion construct was delivered into the lateral ventricle of embryos by *in utero* electroporation [50]. The offspring at p7 or p21 were anesthetized and fixed by vascular perfusion as previously described [51] with Ringer's solution followed by 4% formaldehyde made fresh from paraformaldehyde (Electron Microscopy Sciences) in 0.15 M cacodylate buffer. Brains were removed and placed in the same fixative at 4°C for 1 h for p21 and overnight for p7. In this case we avoided glutaraldehyde in combination with paraformaldehyde due to the increased autofluorescence that occurs with glutaraldehyde. The autofluorescence obscured miniSOG fluorescence and made it impossible to locate transfected neurons in the brain slices for photooxidation. Brains were then sliced to 100 µm sections using a vibratome (Leica). Areas of interest were identified by confocal microscopy. The sections were then postfixed with 2% glutaraldehyde for 30 min, rinsed in cold buffer, blocked, and then photooxidized as described above. Subsequent procedures for EM processing were similar to those described above except the vibratome sections were resin embedded between two liquid release agent coated glass slides (Electron Microscopy Sciences).

### Electron Microscopy

Photooxidized areas of embedded cultured cells were identified by transmitted light and the areas of interest were sawed out using a jeweler's saw and mounted on dummy acrylic blocks with cyanoacrylic adhesive. The coverslip was carefully removed, ultrathin sections were cut using an ultramicrotome, and electron micrographs recorded using a 1200 TEM (JEOL) operating at 80 keV. For tissue sections, one of the glass coverslips was removed using a razorblade and the area of interest identified by transmitted light microscopy. The tissue was removed from the slide, mounted, sectioned, and imaged as above. For electron tomography, 0.5 micron thick sections of cells expressing photooxidized H2B-miniSOG were cut and imaged using a 4000 IVEM (JEOL) operated at 400 keV. Images were tilted and recorded every 2° from ±60° to −60°. The image stack was aligned and reconstructions were obtained using R-weighed back projection methods with the IMOD tomography package. For serial block face scanning electron microscopy, a 3View system (Gatan Inc., Pleasanton, CA) mounted in a Quanta FEG scanning electron microscope (FEI Company, Eindhoven, The Netherlands) was employed. Imaging was performed as previously described [37]. Individual image planes were hand segmented to outline the plasma membrane of the target neuron and denote labeled post-synaptic densities, then thresholded and projected using Amira (Visage Imaging, Germany).

## Supporting Information

Figure S1 Degradation of ADPA by ReAsH, KillerRed, or Rose Bengal (RB) upon irradiation.
(TIF)

Figure S2 Destruction of IFP1.4 by Rose Bengal upon illumination. IFP absorbance (upper left) and fluorescence (lower left) are decreased, proportional to irradiation (540/30 nm) time in the presence of Rose Bengal, which absorbs maximally at 560 nm. IFP absorbance (upper right) and fluorescence (lower right) do not change significantly with the same irradiation time in the absence of Rose Bengal.
(TIF)

Figure S3 Sequence alignment of miniSOG with its parent, the LOV2 domain of AtPhot2. Mutations are highlighted in cyan.
(TIF)

Figure S4 Size exclusion chromatography of miniSOG (red) and its tandem dimer td-miniSOG (blue).
(TIF)

Figure S5 Mass spectroscopy of FMN extracted from miniSOG.
(TIF)

Figure S6 LC/MS of untransfected HEK293 cell lysate spiked with FMN.
(TIF)

Figure S7 LC/MS of untransfected HEK293 cell lysate.
(TIF)

Figure S8 LC/MS of miniSOG-transfected HEK293 cell lysate.
(TIF)

Figure S9 MiniSOG produces EM contrast in labeled organelles and proteins in cells. (A) Adjacent HeLa cells showing differential contrast between photooxidized cells expressing miniSOG tagged alpha-actinin (arrows) versus a non-expressing cell (arrowheads). (B) Adjacent HeLa cells showing differential contrast between a photooxidized cell expressing miniSOG-targeted mitochondria (arrows) versus a non-expressing cell (arrowheads). (C, D) Low and high magnification showing alpha-actinin tagged miniSOG. Bars, 500 nm.
(TIF)

Figure S10 HeLa cells transfected with miniSOG-labeled H2B undergo mitosis. Bars, 2 microns.
(TIF)

Figure S11 High resolution EM reveals presynaptic labeling of SynCAM1 tagged with miniSOG in cultured cortical neurons from randomly selected areas. Scale bars, 500 nm.
(TIF)

Figure S12 Pre- and postsynaptic localization of SynCAM1 and SynCAM2 revealed by miniSOG. (A) Presynaptic localization of SynCAM1-miniSOG in a single neuron forming synapses on itself in micro-island culture, revealed by EM. (B) High resolution EM reveals postsynaptic localization of SynCAM2 labeled by miniSOG in cultured cortical neurons from randomly selected areas. Scale bars, 500 nm.
(TIF)

Figure S13 Stereo pair maximum intensity volume representation of a SynCam2-miniSOG serial block-face scanning electron microscopy reconstruction. The volume consists of 224 image planes (backscatter electron images) recorded at 60 nm intervals in z using 6k × 6 k pixels at 2.5 keV accelerating voltage. Once projected, the image contrast was inverted, with the transfected neuronal processes shown in white. The postsynaptic labeled SynCAM2-miniSOG is shown in blue. Mitochondria in untransfected neurons are also shown. Bars, 2 microns. Movie showing the image stack and 3-dimensional tracing of the transfected neuron reconstruction from SBFSEM shown in Figure S13 (http://login.ncmir.ucsd.edu/~mterada/msog/msog-syncam2b.mp4).
(TIF)

Table S1 MiniSOG does not perturb HEK293 cell growth.
(DOC)

Table S2 FMN is tightly bound in miniSOG.
(DOC)

## Abbreviations

ADPA: anthracene-9,10-dipropionic acid
Cx43: connexin 43
DAB: diaminobenzidine
EM: electron microscopy
FA: focal adhesion
FAD: flavin adenine dinucleotide
FM: fluorescence microscopy
FMN: flavin mononucleotide
GFP: green fluorescent protein
H2B: histone 2B
HRP: horseradish peroxidase
IFP: infrared fluorescent protein
LOV: light-oxygen-voltage
miniSOG: small singlet oxygen generator
NGL: netrin-G ligand
SBFSEM: serial block-face scanning electron microscopy
SEM: scanning electron microscopy
SynCAM: synaptic cell-adhesion molecules
