# Supplementary figures and images for: A Genetically Encoded Tag for Correlated Light and Electron Microscopy of Intact Cells, Tissues, and Organisms
Source: PLoS Biol. 2011 Apr 5;9(4):e1001041. doi: 10.1371/journal.pbio.1001041 (PMC3071375; doi:10.1371/journal.pbio.1001041)

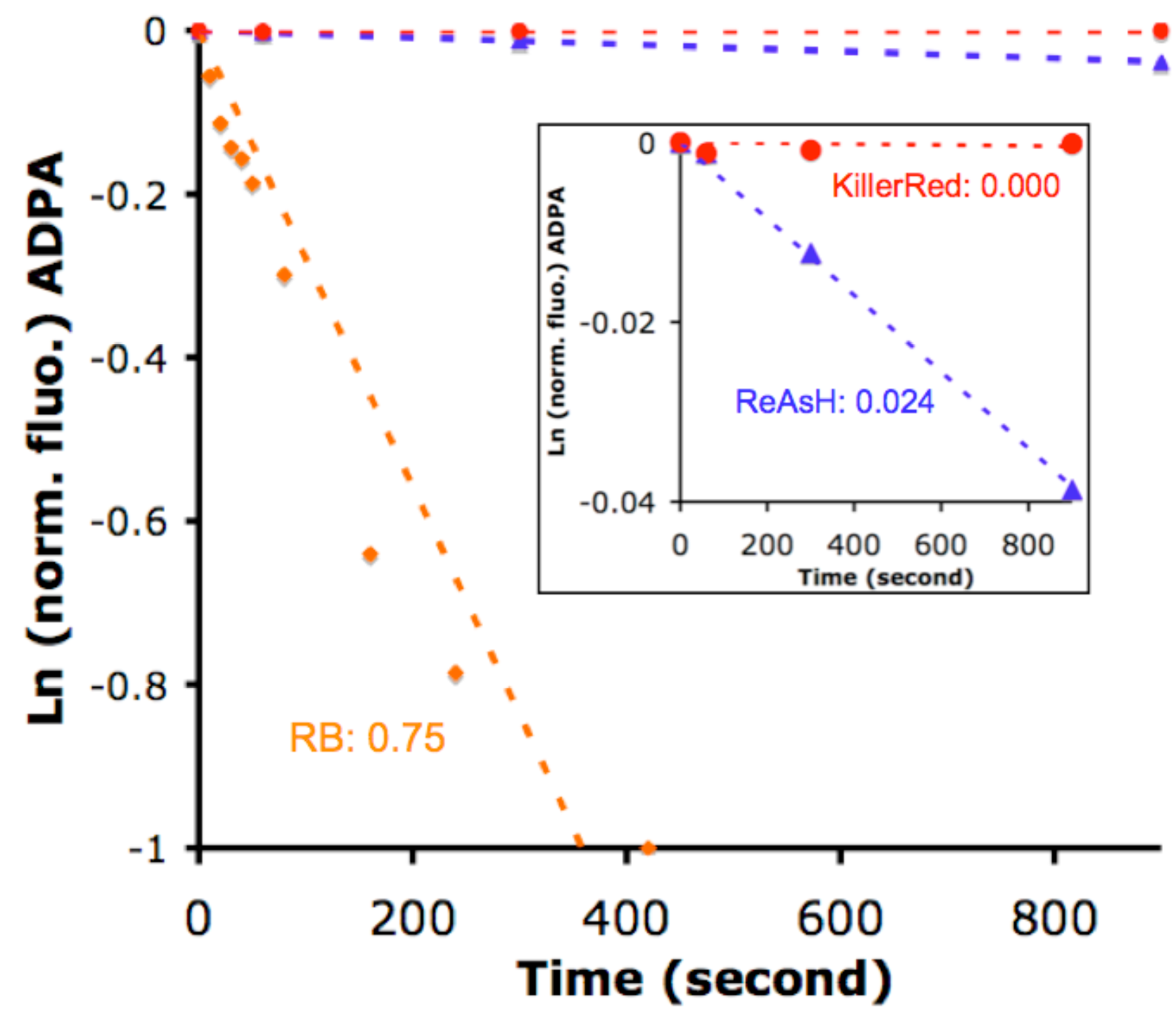

Supplement: Figure S1 — Degradation of ADPA by ReAsH, KillerRed, or Rose Bengal (RB) upon irradiation. (TIF) [file pbio.1001041.s001.tif]

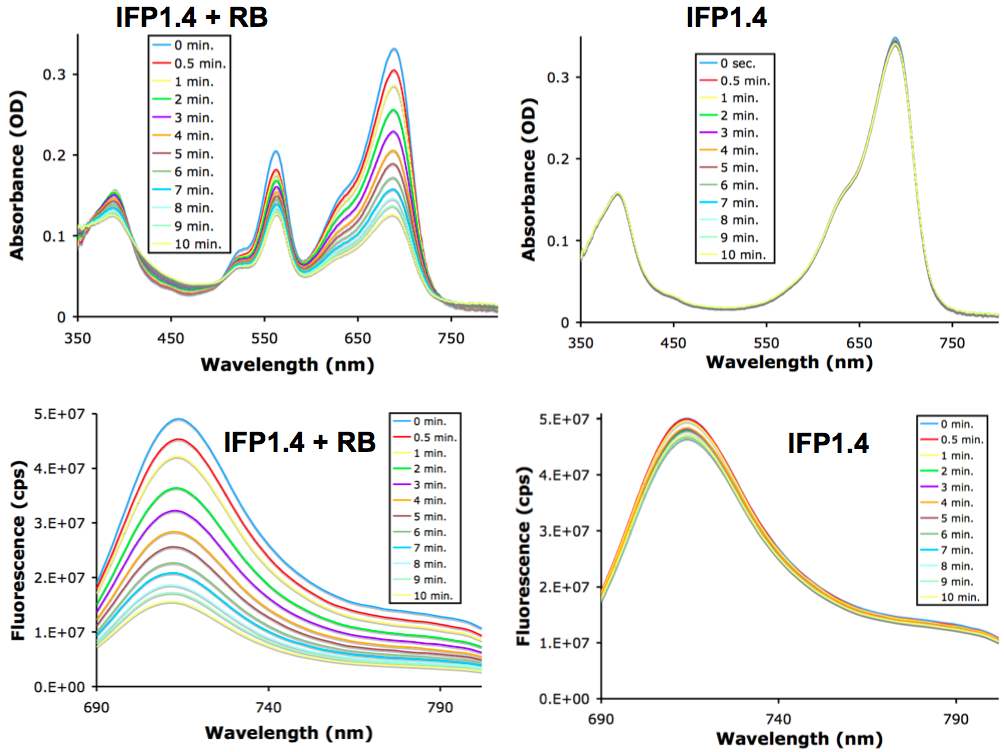

Supplement: Figure S2 — Destruction of IFP1.4 by Rose Bengal upon illumination. IFP absorbance (upper left) and fluorescence (lower left) are decreased, proportional to irradiation (540/30 nm) time in the presence of Rose Bengal, which absorbs maximally at 560 nm. IFP absorbance (upper right) and fluorescence (lower right) do not change significantly with the same irradiation time in the absence of Rose Bengal. (TIF) [file pbio.1001041.s002.tif]

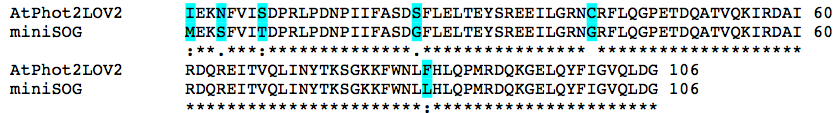

Supplement: Figure S3 — Sequence alignment of miniSOG with its parent, the LOV2 domain of AtPhot2. Mutations are highlighted in cyan. (TIF) [file pbio.1001041.s003.tif]

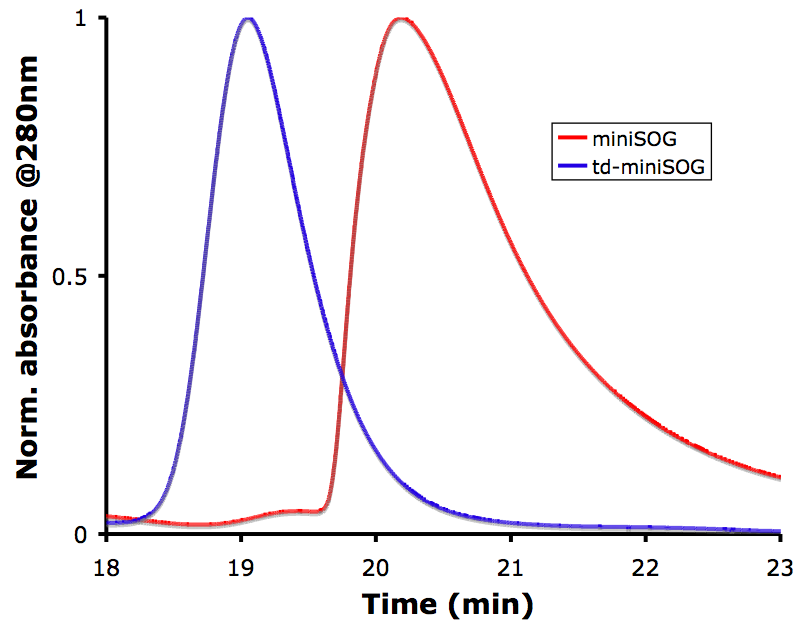

Supplement: Figure S4 — Size exclusion chromatography of miniSOG (red) and its tandem dimer td-miniSOG (blue). (TIF) [file pbio.1001041.s004.tif]

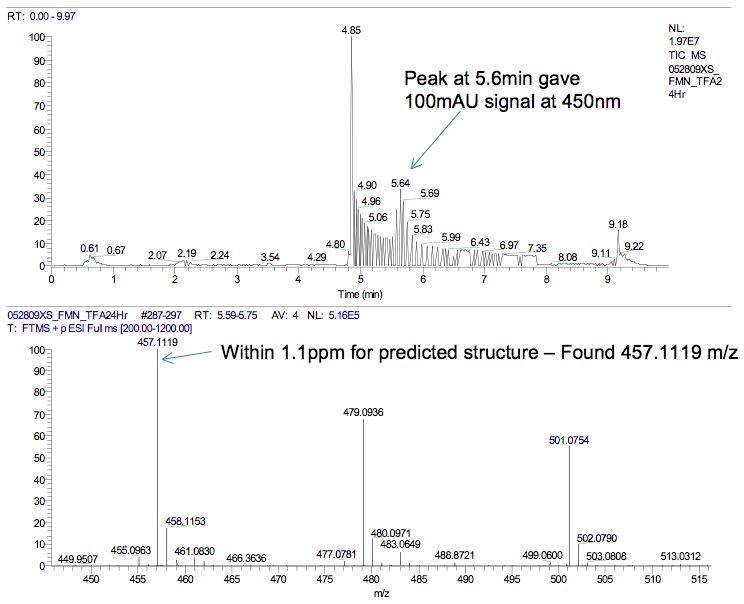

Supplement: Figure S5 — Mass spectroscopy of FMN extracted from miniSOG. (TIF) [file pbio.1001041.s005.tif]

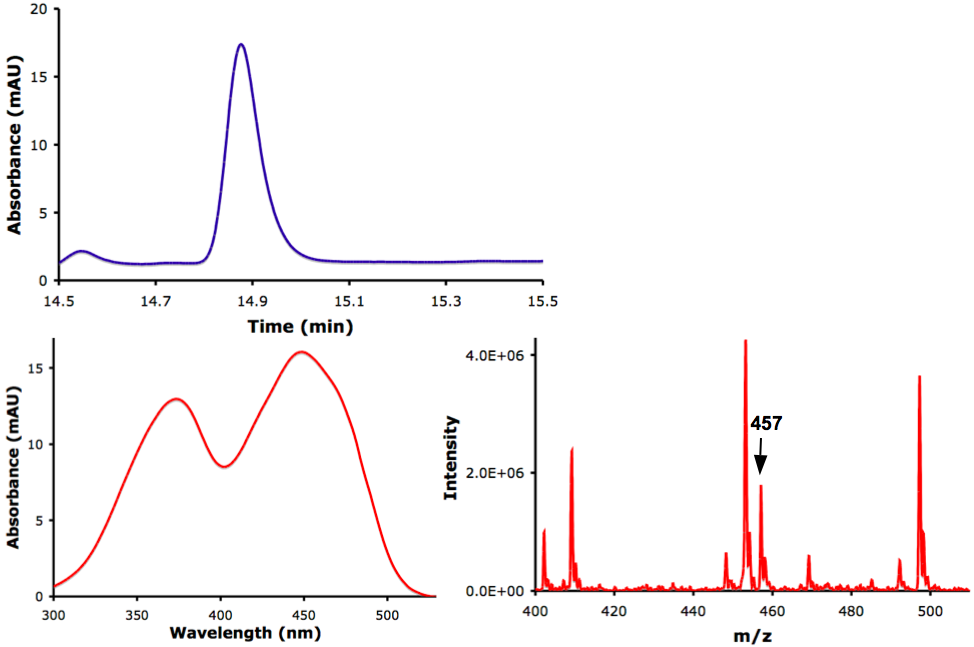

Supplement: Figure S6 — LC/MS of untransfected HEK293 cell lysate spiked with FMN. (TIF) [file pbio.1001041.s006.tif]

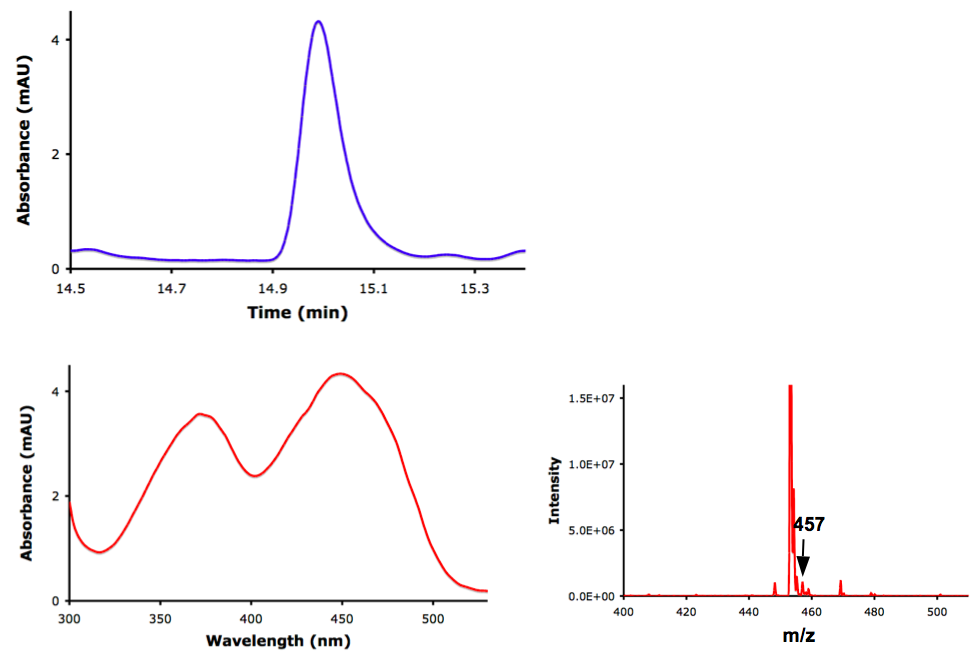

Supplement: Figure S7 — LC/MS of untransfected HEK293 cell lysate. (TIF) [file pbio.1001041.s007.tif]

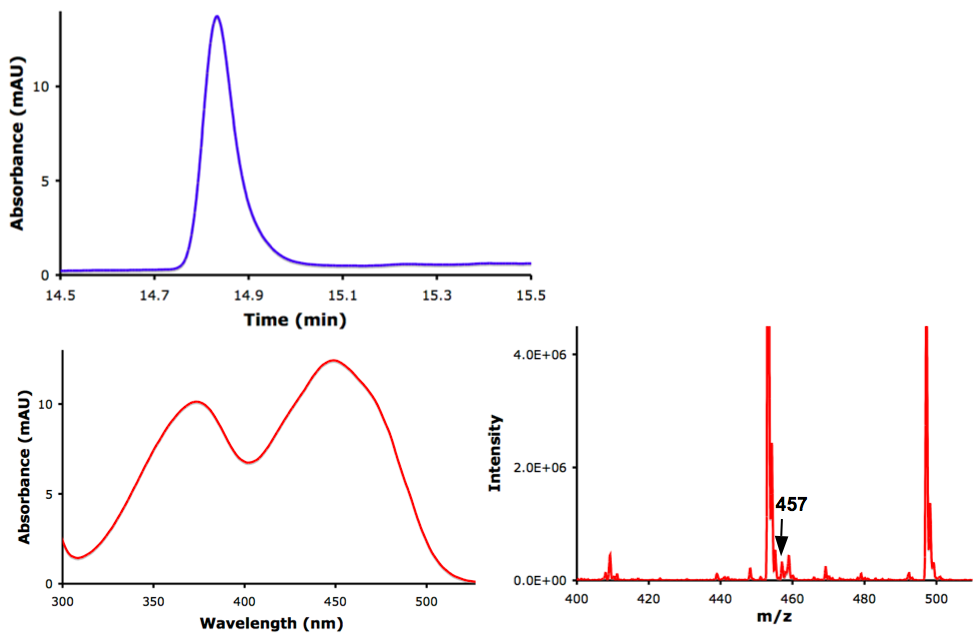

Supplement: Figure S8 — LC/MS of miniSOG-transfected HEK293 cell lysate. (TIF) [file pbio.1001041.s008.tif]

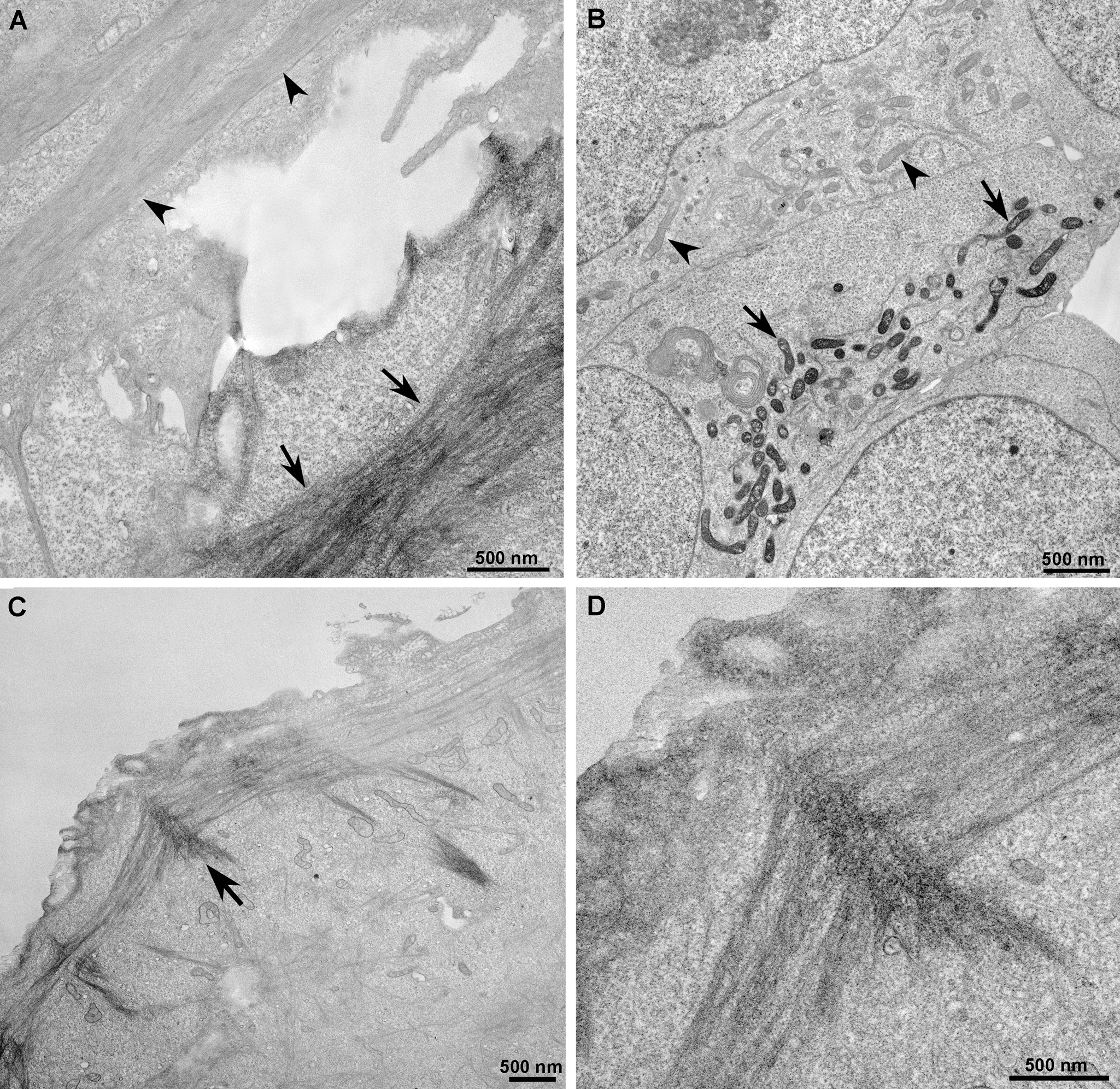

Supplement: Figure S9 — MiniSOG produces EM contrast in labeled organelles and proteins in cells. (A) Adjacent HeLa cells showing differential contrast between photooxidized cells expressing miniSOG tagged alpha-actinin (arrows) versus a non-expressing cell (arrowheads). (B) Adjacent HeLa cells showing differential contrast between a photooxidized cell expressing miniSOG-targeted mitochondria (arrows) versus a non-expressing cell (arrowheads). (C, D) Low and high magnification showing alpha-actinin tagged miniSOG. Bars, 500 nm. (TIF) [file pbio.1001041.s009.tif]

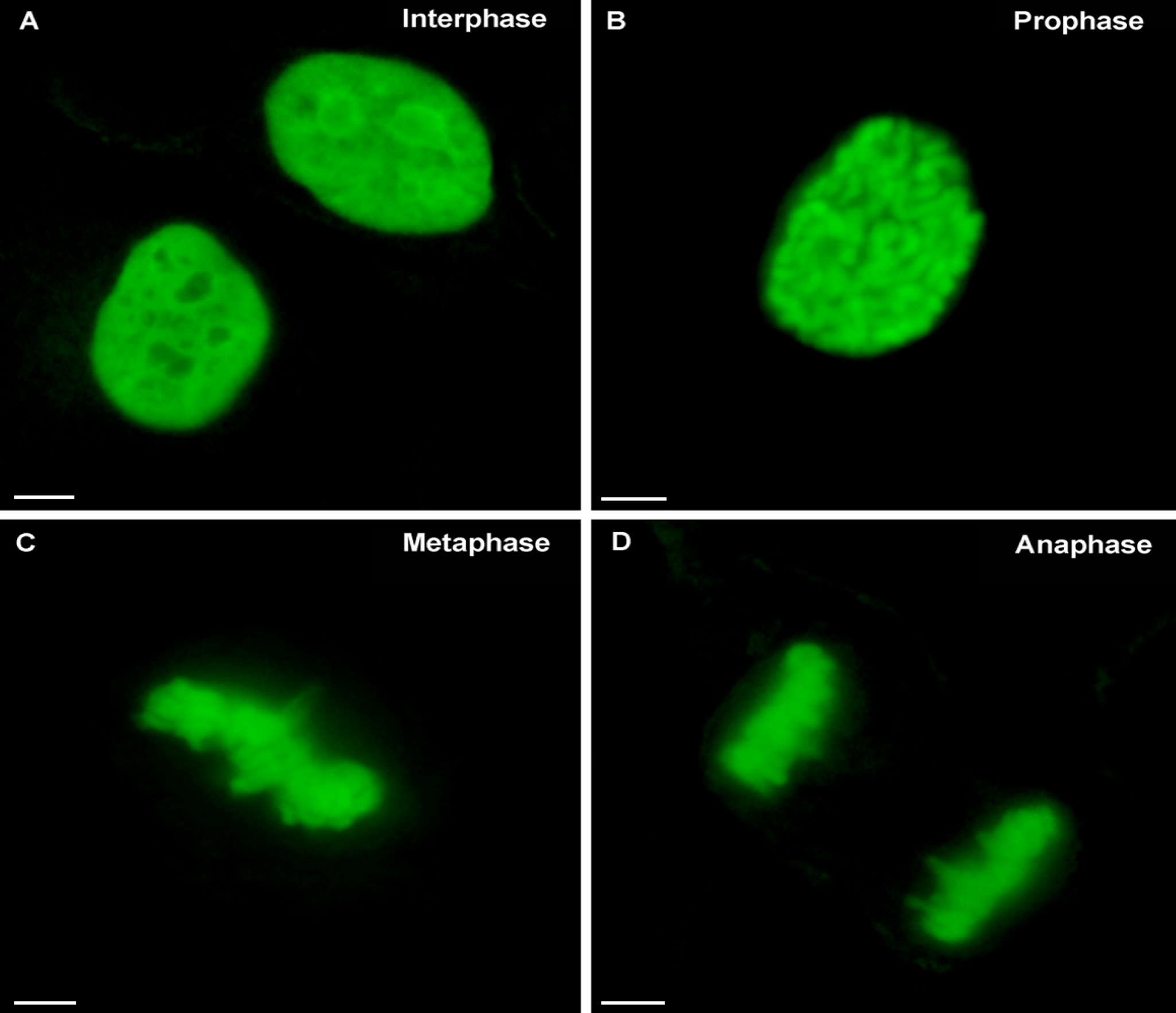

Supplement: Figure S10 — HeLa cells transfected with miniSOG-labeled H2B undergo mitosis. Bars, 2 microns. (TIF) [file pbio.1001041.s010.tif]

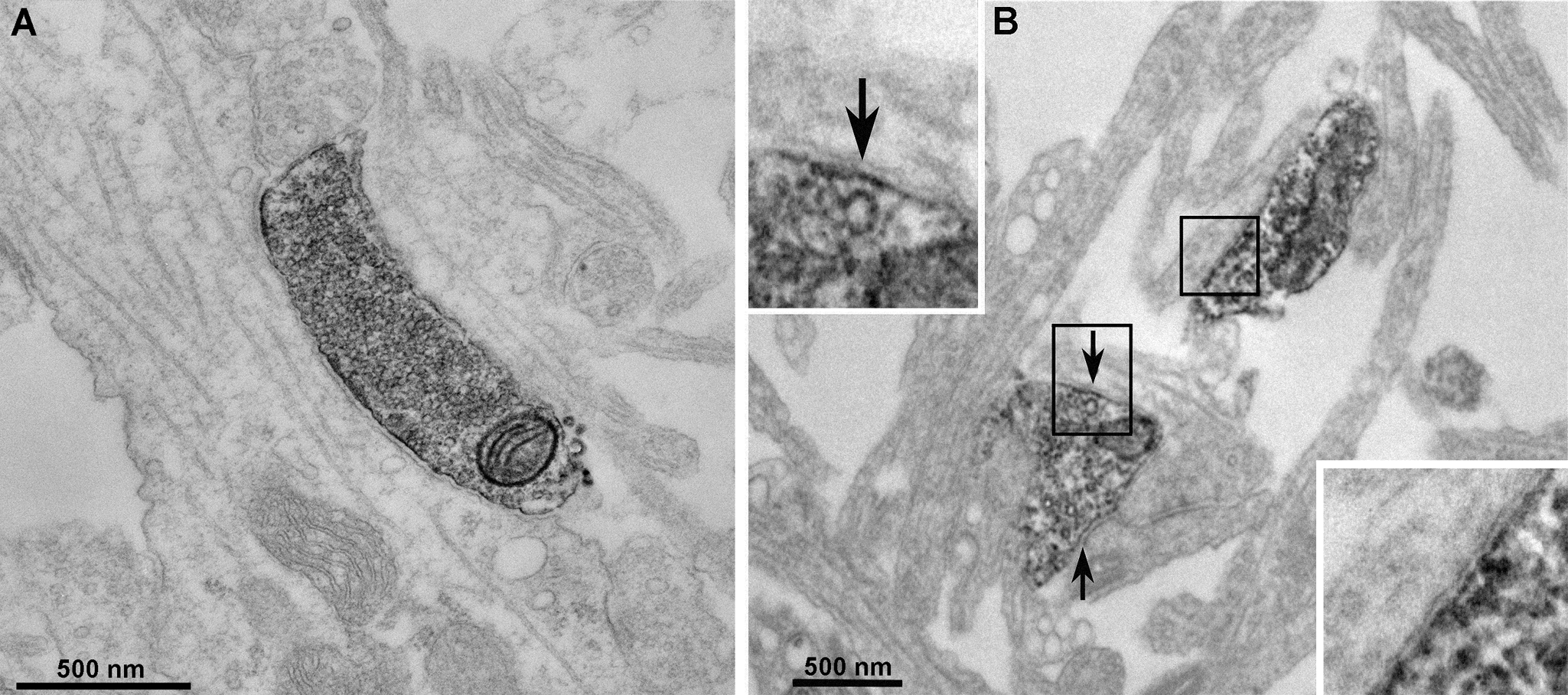

Supplement: Figure S11 — High resolution EM reveals presynaptic labeling of SynCAM1 tagged with miniSOG in cultured cortical neurons from randomly selected areas. Scale bars, 500 nm. (TIF) [file pbio.1001041.s011.tif]

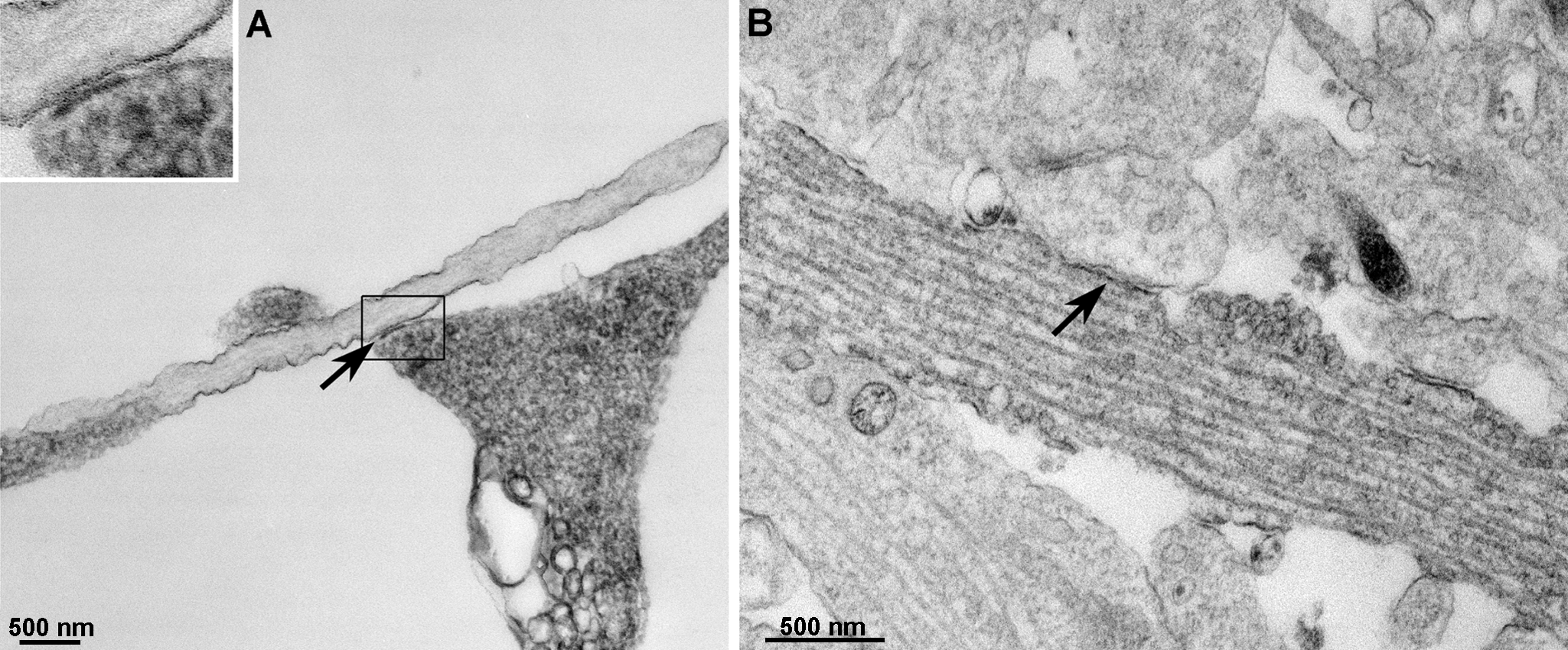

Supplement: Figure S12 — Pre- and postsynaptic localization of SynCAM1 and SynCAM2 revealed by miniSOG. (A) Presynaptic localization of SynCAM1-miniSOG in a single neuron forming synapses on itself in micro-island culture, revealed by EM. (B) High resolution EM reveals postsynaptic localization of SynCAM2 labeled by miniSOG in cultured cortical neurons from randomly selected areas. Scale bars, 500 nm. (TIF) [file pbio.1001041.s012.tif]

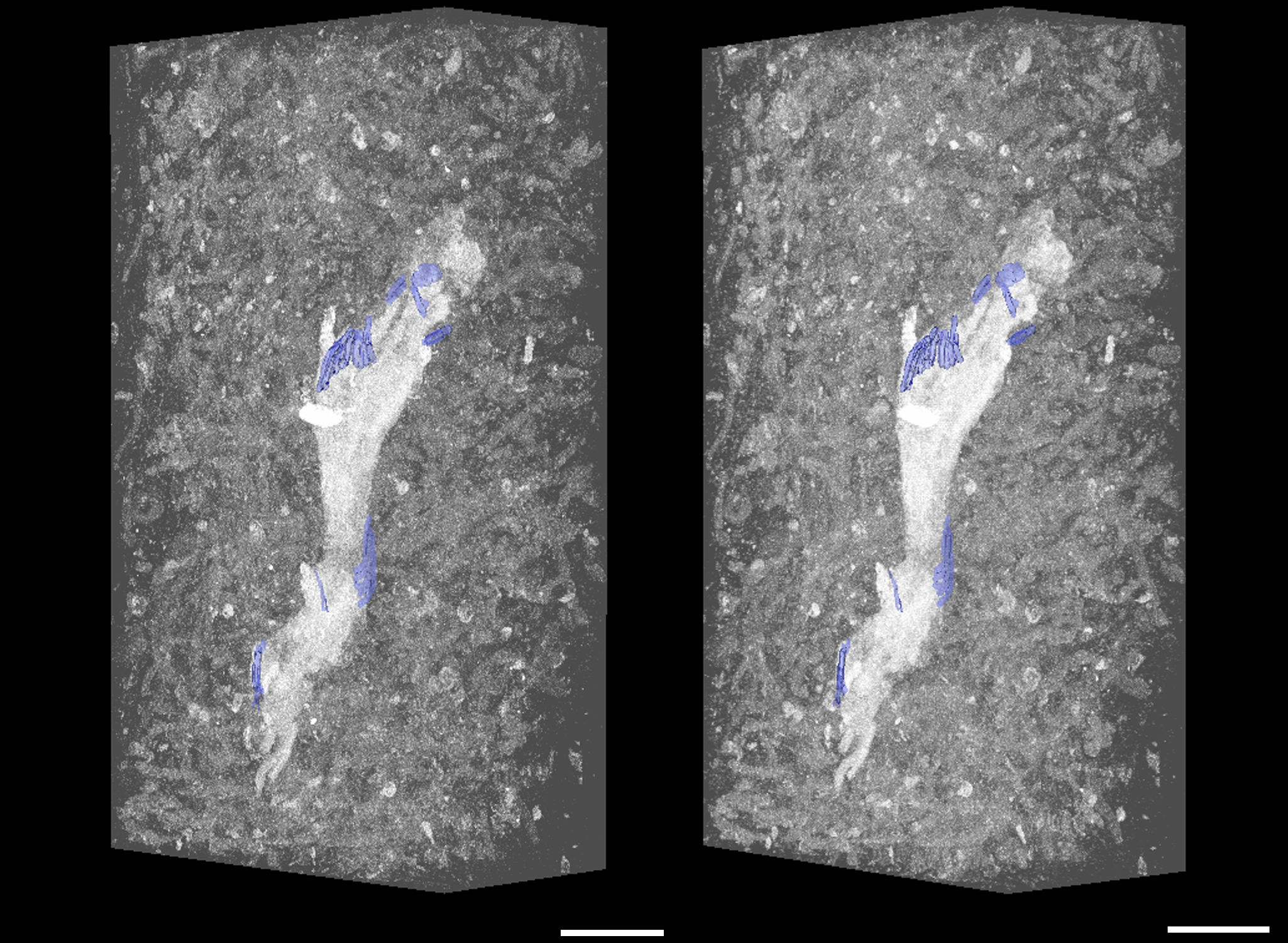

Supplement: Figure S13 — Stereo pair maximum intensity volume representation of a SynCam2-miniSOG serial block-face scanning electron microscopy reconstruction. The volume consists of 224 image planes (backscatter electron images) recorded at 60 nm intervals in z using 6k × 6 k pixels at 2.5 keV accelerating voltage. Once projected, the image contrast was inverted, with the transfected neuronal processes shown in white. The postsynaptic labeled SynCAM2-miniSOG is shown in blue. Mitochondria in untransfected neurons are also shown. Bars, 2 microns. Movie showing the image stack and 3-dimensional tracing of the transfected neuron reconstruction from SBFSEM shown in Figure S13 (http://login.ncmir.ucsd.edu/~mterada/msog/msog-syncam2b.mp4). (TIF) [file pbio.1001041.s013.tif]
