# Supplementary material for: A Genetically Encoded Tag for Correlated Light and Electron Microscopy of Intact Cells, Tissues, and Organisms
Source: PLoS Biol. 2011 Apr 5;9(4):e1001041. doi: 10.1371/journal.pbio.1001041 (PMC3071375; doi:10.1371/journal.pbio.1001041)
Supplement: Table S1 — MiniSOG does not perturb HEK293 cell growth. (DOC) [file pbio.1001041.s014.doc]

**Table S1** **Growth rate of HEK293 cells.**

|  |  | Cell number (10^3^) |  |
| --- | --- | --- | --- |
|  | Day 0 | Day 1 | Day 2 |
| miniSOG | 200 | 390 | 940 |
| EGFP | 200 | 400 | 920 |
| untransfected | 200 | 400 | 930 |

*transfection efficiency > 90%
