# Supplementary material for: A Genetically Encoded Tag for Correlated Light and Electron Microscopy of Intact Cells, Tissues, and Organisms
Source: PLoS Biol. 2011 Apr 5;9(4):e1001041. doi: 10.1371/journal.pbio.1001041 (PMC3071375; doi:10.1371/journal.pbio.1001041)
Supplement: Table S2 — FMN is tightly bound in miniSOG. (DOC) [file pbio.1001041.s015.doc]

**Table S2** **miniSOG dialysis.**

| Dialysis time (hrs) | [apo]*[FMN]/[holo] (10^-12^M) |
| --- | --- |
| 24 | 123.8 ± 5.7 |
| 48 | 173.6 ± 5.3 |
| 72 | 170.3 ± 8.4 |
